# From Spark to Flame -- Radical Innovations from Cataclysmic Events in Medical Education

**DOI:** 10.15694/mep.2020.000132.1

**Published:** 2020-06-24

**Authors:** Jessica Foulds, Martha Burkle, Lyn K. Sonnenberg

**Affiliations:** 1 University of Alberta

**Keywords:** Medical education, Undergraduate medical education, Postgraduate medical education, Health Professions Education, Educational Technology, Clinical teaching, Virtual learning, LMS (learning management system), COVID-19

## Abstract

This article was migrated. The article was marked as recommended.

We all knew it was coming. We just didn’t realize it would all come at once. No, we are not talking about the zombie apocalypse, but rather the emergence of virtual teaching and virtual healthcare delivery pervading every aspect of life as we now know it. In the context of COVID-19 and marked shifts in how and where we teach medical learners, the staggering number of new ideas, adaptations, and innovations has been inspiring. This game-changing pandemic is a spark, a lightning bolt if you will, that has created solutions, where previous barriers may have been in virtual teaching and healthcare provision. It is impossible to even consider going “back to normal”, as they say. We believe the torrent of ideas and possibilities for medical education, brought by COVID-19, cannot and should not be stopped. We explore the nuances of virtual teaching and virtual care and seek readers to consider what their actionable frameshift can mean for medical education in their teaching realm moving forward. We believe that this is the time to innovate: the time to radically change our traditional medical education practices. To sustain these innovations, institutional support, participant buy-in, and assessment and outcome data will be invaluable to harness these new opportunities.

## The emergency framework – an opportunity for innovation

Winds of change would be an understatement. A cataclysmic change to teaching occurred with COVID-19, and before we knew which way was up, virtual teaching was what we had to do. Virtual teaching refers to the use of remote video conferencing software, web-based learning management systems, and other digital technology, to teach students from a distance. With the abrupt shift to online distance learning amidst COVID-19, there has been a steep learning curve for educators. The disruptions, cancellations, modifications, and anxieties have been numerous, but they offer an opportunity to foster creativity, innovation, and more significant evolution in medical education strategies. Many local, regional, and national institutions and associations have developed tips to support clinicians during this time (for example ( Royal College of Physicians and Surgeons of Canada: Virtual Teaching Resources, 2020). Clinical experiences have undergone significant shifts to virtual care and the learning environment for medical learners is markedly different ( Wosik *et al*., 2020). Amidst a forced function of change, there no doubt will be a lasting legacy of increasing integration of virtual teaching and virtual care for medical learners. As William Osler once said “The future is today” ( Osler, 1913), and we have the opportunity to leverage the scaffolding of an emergent framework and create lasting, innovative, improved cultural shifts in medical education.

## A model for teaching innovation – when there is no time for planning: A call to action

Using a “flame and fire” metaphor, we believe that the arrival of COVID-19 has been the trigger for the innovation that medical education has been awaiting for decades. The previously optional use of virtual technologies to support teaching and learning has now become a requirement to continue to reach our learners, wherever they are. But in order for the spark to remain alive (even if the situation of “back to normal” is possible, which we believe is not), radical innovations need institutional support to become modus operandi, the new ways in which we teach and learn across institutions ( Zellweger and Moser, 2007). We know that early adopters of technology or ‘lone rangers’ have embraced technology innovation since the start. However, in order for technology to be sustainable, there is a need for all (or most) faculty to get involved, take the risk of innovation, and be bold. And, in order not to smolder out, educational innovation, as with any other type of innovation, needs leadership support, to find its way from being only a risky idea, to becoming part of the institution’s strategic vision and plan.

**Figure 1.**
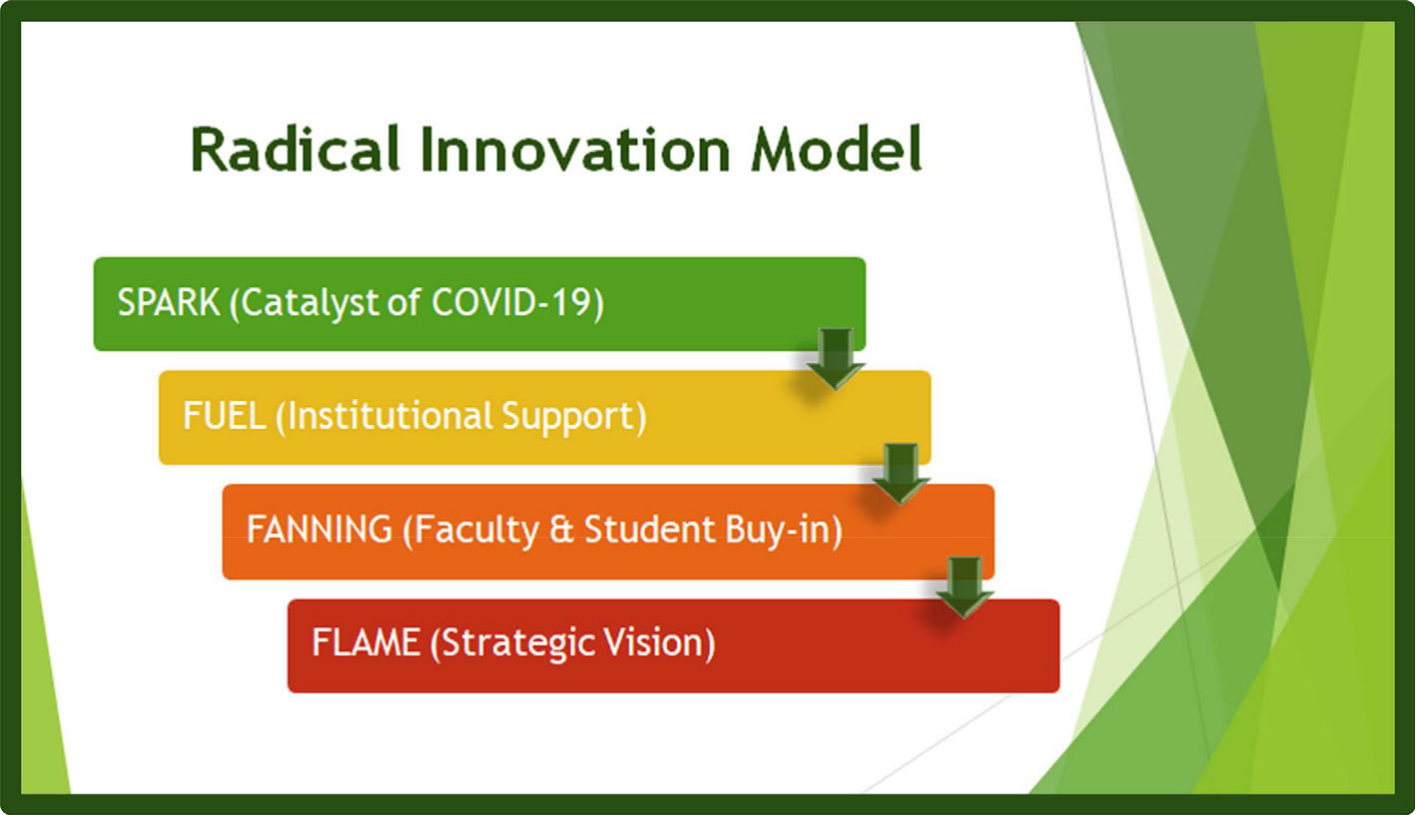
Radical innovation model for teaching and learning

## Virtual teaching - barriers and solutions

Educators may feel that in this marked shift of medical education delivery they have been forced to ignore tenets of curriculum development, or the pedagogy of online learning (including the principles, methods, and practice of teaching and learning ( Chen *et al*., 2019)), acting with haste and without consideration of a learner-centred lens. An integrative review, published prior to any COVID-19 ramifications, identified several barriers, and solutions, for transitions to online medical education ( O’Doherty *et al.*, 2018). In this integrative review, O’Doherty and colleagues identified four core themes: skill deficits, time and infrastructure resources, institutional strategies and support, and attitude. Solutions offered included engagement, collaboration, looking at what has already been put in place (and maybe had outcomes measured!), and self-compassion. These perceived barriers excitingly have many more solutions in our shift to virtual teaching amidst COVID-19.

Academic institutions, and medical education collaborative groups, have responded with great supports for scaling up online teaching and learning skills. The numbers of tips, tricks, lessons learned, online “living documents”, active social media threads, and forums are growing by the day. Knowing you are not alone in your transition to virtual teaching, and that there are free open access ‘meducation’ (FOAM) resources ( Nickson & Cadogan, 2014) available to you, can be a good place to start.

## From lecturing to facilitating - the virtual classroom

*“I cannot teach anyone anything. I can only make them think”* Socrates

For years, if not for decades now, universities have only thought about the possibility of moving their classroom spaces to the virtual environment. The old idea of the importance of the classroom as a brick and mortar institution, where faculty meet their students, has influenced the way policies are written at the university administration level, requiring student attendance, faculty training oriented to lecturing, classroom allocation, and campus infrastructure. For a long time the so-called “lone-rangers” - innovative faculty who test the use of technologies to innovate their teaching (Bates, 2000) - have been operating on their own, bringing innovative models inside and outside the classroom and creating ‘pockets of innovation’ across disciplines and programs.

It is only now, when triggered by a pandemic, universities have been literally pushed to catch up with teaching innovation and start using synchronous and asynchronous delivery supported by technologies. For those of us advocating for years for the adoption of flexible, student-oriented strategies for learning remotely, it seems our time has finally arrived.

When considering the use of virtual teaching strategies, consideration of synchronous or asynchronous delivery is an important distinction. Do the learner and the facilitator need to be present simultaneously? If so, what is their role? The traditional teacher-centred lecture can happen synchronously with learners, but if there is no role for questions and answers, interaction, or knowledge application and feedback, did it need to be live? These are existential questions that have come to light during COVID-19, but hold true for any teaching and learning encounter.

Active learning strategies refer to any instructional method that engages students in the learning process ( McCoy, Pettit, Kellar, & Morgan, 2018). Some examples of these methods include flipped classrooms, case-based learning, problem-based learning, brainstorming or “fishbone”, and jigsaw techniques ( Hew & Lo, 2018). Fostering teamwork and interaction between learners, to acquire and apply knowledge, are the goals of each of these different strategies ( Torralba & Doo, 2020). Some of these strategies require multiple facilitators and so can be limiting based on the time and availability of facilitator expertise. Breakout rooms can function as small group rooms and allow for these strategies in a virtual learning environment.

For synchronous learning encounters, there are several small modifications you can make to increase interactivity and enhance experiential learning, many of which have been learned from distributed medical education models, established well before the rapid COVID-19 pivot ( Bin Mubayrik, 2020; Wong *et al*., 2012). Engaging your learners and asking for them to create an answer or solution instead of providing the answer to your question is an approach often overlooked as an active teaching strategy! The key to this in a virtual teaching environment is that you must patiently wait for their answer; if you ask and no one speaks up, encourage the use of chat or text features. The use of variable changes, or “what if” questions can be a simple modification to case examples in checking for understanding with your learner audience ( Rana & Burgin, 2018). You can also use media to promote learner engagement (technology enhanced active learning), like online polling for quizzes, audience “temperature checks”, and serious games, which have been noted to increase learner reported self-attentiveness ( Castillo *et al.*, 2020). So many of us are more than aware of what it is like to sit in long, endless, remote meetings; don’t be afraid to try something to help your learner shake the cobwebs off!

## Consolidating a learning management system - the role of infrastructure

But what happens when you walk away from the live, synchronous teaching environment? How do you keep learners engaged and onboard with your vision? Learning Management Systems (LMS) are web-based platforms that allow faculty and students to share learning materials, complete assignments, submit quizzes and tests, and interact with each other inside and outside the classroom space ( Al-Busaidi & Al-Shihi, 2012). Beyond being a repository for attachments and documents, LMS create the virtual infrastructure that allows the delivery and management of course content, identifies and assesses learners, and tracks their progress. Their use offers a curated experience that allows the learner to explore content, review key references and assignments at their own pace, and interact with peers, all while learning from each other ( Back *et al*., 2016). It does require more of the instructor’s time at the beginning to prepare a course, but once it has been developed and uploaded, it is well worth the time invested. Learner progress and student engagement can be monitored more readily than in a large classroom setting (that none of us have access to in pandemic times). There is no way the gallery manager could give a personal guided interpretation live and in-person to every visitor; but the flow and layout of the art, and the accompanying thought-provoking questions? All these are offered by LMS.

Since the arrival of LMS to higher education in 1991, they have become a powerful tool to support teaching and learning at a distance ( Anderson & Dron, 2017). Numerous LMS options are available including Blackboard, Desire to Learn, eCollege and Moodle, which is open source, to mention a few. A virtual learning environment should be planned and organized in advance, from A to Z. Centres supporting the use of technologies for teaching and learning (such as information service and technology, centres for teaching and learning, and educational technologies) exist to help faculty transition their courses to LMS, or request instruction on how to use the system to its greater potential, such as assessment delivery ( Gummesson, Hellmark, & Edgren, 2017). Consult with an expert, chat with your peers, learn from what others are doing. LMS will radically change the way you teach, in your favour, but as mentioned, a bit of a learning curve is required (Bates, 2020).

Once you embrace LMS, the world is your oyster. There are a whole host of possibilities for interaction with content and learners that these platforms provide. You should take full advantage of these features. Using your LMS as only a reading repository is a waste of time and resources, and your students will not feel engaged. In the current pandemic environment, think about how to motivate critical thinking amongst your students by designing intelligent questions to pose in the discussion forums, or creating multiple formative assessment opportunities for your students to evaluate themselves as they go through the teaching material. And don’t forget to check their progress. You cannot be replaced by LMS or artificial intelligence, as exciting as these developments are in higher education; your availability and mentorship of your learners is irreplaceable.

Be mindful of your learners’ connectivity and bandwidth. Some of them will have strong data access through their internet connections, but a large number of them, who may be connecting from more remote communities, may not. Think about this when you plan to upload large videos to your LMS or choose interactive virtual platforms that require lots of bandwidth ( Minasian-Batmanian, 2002). Also, consider their cognitive bandwidth. Avoid long activities and lectures; remote learners are surrounded by lots of distracting factors. Small capsules of information, supported by videos and discussion forums, work much better than an hour long video recorded lecture ( Galway, *et al.*, 2014).

As much as LMS may seem like new and foreign technology to many, the principle of having infrastructure and scaffolding to facilitate teaching, while curating a valuable learning experience, is likened to the role clinical preceptors play in the clinical learning environment.

## Delivering patient care with resident physicians and learners - in the virtual world

Virtual care is here to stay and is an essential skill for medical learners today. Unfortunately, our apprenticeship model of learning has left clinical preceptors in the dark around exactly how to embrace this new model of healthcare delivery. For those clinicians who have embraced the shift to virtual care, most will note that having a learner integrated into the visit is a lot less challenging than initially perceived. Many will even note the enriched observations they are able to provide learners, as preceptors may now observe entire encounters, and patients seem content and focused on the learner in the lead role, instead of deferring to the senior clinician. Learners, equally, are embracing both the technology and the feedback, even if the clinical opportunities are fewer in some settings. Optimizing learner involvement can occur in a number of ways, but many of which you already do in traditional clinical encounters: establish expectations, establish the degree of supervision/support the learner requires, make a plan for the day, and provide small, actionable pieces of feedback! From a technology perspective, try using “breakout rooms” as individual patient/clinic rooms, having patients arrive in the virtual waiting room, where they can then be assigned a room. As the clinical preceptor, you can go between rooms to see patients, and can even meet in the “team” breakout room to speak with a learner, if needed. If you view technology as your friend, and think outside the box, there are no limits to what can be facilitated.

And that has been made clear by others who have recently published on how to support learners in the virtual world. A great place to start is with The College of Family Physicians of Canada’s Tips for Supervising Family Medicine Learners Providing Virtual Care guide, which highlights the need to check the learner’s needed level of supervision a priori ( Oandasan, Cavett, Singer, & Wolfrom, 2020). It also provides ten questions to consider when reviewing virtual cases with learners, such as “is the learner aware of the limitations of the technology used?” Telehealth essentials are overviewed in another easy to use document to help you set up the telehealth basics in general, with questions learners can ask regarding virtual healthcare consent and what to do in an emergency, if your site does not already have their own recommendations in place ( Arsenault, *et al.*, 2020). You do not always need to reinvent the wheel.

## Looking to the future

Considering all the other competing demands during a pandemic, the rapidity in which content has shifted to virtual delivery is staggering. How these innovations, triggered by COVID-19, are embraced and celebrated will be exciting to see unfold. We envision that every member of the learning community (faculty, students, staff and the University) will benefit from a radical transformation of our roles in the recovery stage of this pandemic. We all have heard from colleagues working in our faculties the desire for things going “back to normal”, and we highlight that ‘normal’ need not mean going back to the traditional one-to-many teaching model or assess-by-assumption apprenticeship clinical model. We would like to see that ‘normal’ is radically affected by how teaching and learning is happening today.

This is the time to take risks and explore how social media and other new technological platforms can help us in the innovation of our teaching, not merely how we can use technologies to continue teacher-centred lecturing or apprenticeships ( Bahner *et al*., 2012). Development of more student-centred pedagogies when courses are prepared, providing space and opportunities for problem solving, considering our learner’s geographical location, their bandwidth (cognitively and their internet access), and how best we support their learning, are all essential considerations. COVID-19 has caused us to rethink how and what we do as medical educators, now and moving forward. And we shall be better for it!

## Take Home Messages

• Now more than ever, there is help, locally or online, to help enhance your virtual teaching
• Socrates can be your home-boy! Remote interactions with learners require intentionality, and can be as simple as asking for the why behind your observation
• Learning management systems offer an opportunity to curate an educational experience; consider what role they can play for your course delivery
• Leverage technology to enhance clinical learning, even if it is to make the virtual clinic feel more traditional
• Any radical innovation, teaching or otherwise, requires ongoing fuel and fanning for a sustained flame

## Notes On Contributors

**Jessica L. Foulds**, MD, FRCPC, is an Assistant Professor in the Department of Pediatrics and the Assistant Program Director of the General Pediatrics Residency Program, Faculty of Medicine and Dentistry, at the University of Alberta. She enjoys being an optimist and harnessing her enthusiasm into medical education and curriculum development.

**Martha Burkle**, PhD, is the Development Collaborations Coordinator for the Faculty of Medicine & Dentistry, at the University of Alberta. Her research interests lie in the area of the use of information and communication technologies for medical education, for poverty alleviation, and for social development.

**Lyn K. Sonnenberg**, MD, MEd, MSc, FRCPC, is the Associate Dean, Educational Innovation & Academic Technologies for the Faculty of Medicine & Dentistry at the University of Alberta, and an Associate Professor in the Department of Pediatrics. She believes that medical learners are the leaders of today and that technology is only one tool in the medical educator’s toolbox.
