# Supplementary material for: From Spark to Flame -- Radical Innovations from Cataclysmic Events in Medical Education
Source: MedEdPublish (2016). 2020 Jun 24;9:132. [Version 1] doi: 10.15694/mep.2020.000132.1 (PMC10702677; doi:10.15694/mep.2020.000132.1)
Supplement: Peer review history

# Reviewer report: Reviewer response for version 1

**DOI:** 10.21956/mep.20107.r30997

**Published:** 2020-07-04

**Authors:** Virginia Randall

**Affiliations:** 1 Uniformed Services University of the Health Sciences

This review has been migrated. The reviewer awarded 4 stars out of 5 I found this article incredible supportive of our abrupt transition to the virtual platform for medical education. The authors quite correctly point out that virtual teaching/learning has many advantages. At my institution, we have students scattered across the US, and the virtual platform allows us to connect them for group discussions that both convey content, but allow them to experience supportive learning derived from the group.

Reviewer Expertise:

NA

No decision status is available

# Reviewer report: Reviewer response for version 1

**DOI:** 10.21956/mep.20107.r30996

**Published:** 2020-06-26

**Authors:** P Ravi Shankar

**Affiliations:** 1 American International Medical University

This review has been migrated. The reviewer awarded 4 stars out of 5 This is an interesting article examining the rapid changes in teaching-learning which have occurred primarily due to the pandemic. The authors also briefly talk about telemedicine and student involvement in the consultation. How medical school will look like post-COVID is also important to consider though predicting the future is a difficult task. There is no doubt that medical education will be changed in many aspects. Lectures can be delivered online and tasks not requiring interaction and active learning can be shifted online. This provides students the ability to access learning at their own convenience. An issue to consider is that in many crowded, Asian countries students may not have the physical space available in their homes to engage in un-interrupted study. Medical schools may need to provide the physical space to students to ‘study online’ as soon as conditions permit. Also, I believe that as younger students just out of high school and due to the nature of their school education South Asian students may have less self-regulated learning abilities. Teleconsultation may be an option patients may prefer for some conditions especially as they can avoid the commute and the crowds at the clinic or hospital. Having students ‘sit in’ on the consultation is useful. The camaraderie and social interactions of medical school are difficult to create online. I am proficient with online resources but given the choice I would prefer to have a coffee with my friends in the college cafeteria. The physical campus, the physical interactions, and the physical resources are an important part of medical school for most students. During the ongoing pandemic these may be off limits to ensure student safety. For a few months students may be willing to stay away from campus but eventually education will be poorer if we are not able to offer these ‘physical resources’ to the students. Institutions offering their courses online during the pre-COVID era, have for long had a ‘questionable reputation’ due to a variety of reasons. There were some institutions offering medical and health science degrees online which ceased to operate without allowing their students to complete their education. Many medical councils continue to not recognize medical education offered through online modes. What percentage of education can be offered online will be a matter for debate and discussion once a certain degree of physical learning is possible. There is no doubt that online methods can make an important contribution to medical education but in my opinion like the medical curriculum in general, once conditions stabilize the extent and breadth of online learning is something individual countries, regions and medical schools may have to decide on.

Reviewer Expertise:

NA

No decision status is available

# Reviewer report: Reviewer response for version 1

**DOI:** 10.21956/mep.20107.r30998

**Published:** 2020-06-25

**Authors:** sathyanarayanan varadarajan

**Affiliations:** 1 SRM Medical College, SRM University

This review has been migrated. The reviewer awarded 5 stars out of 5 In this excellent article, the authors have explored creatively , the nuances of virtual teaching and virtual care. They’ve ignited a spark in our minds for innovation in medical education calling into action with a "flame and fire" metaphor and seek readers to consider this time of crisis to radically change traditional medical education practices. Throughout the article, they inspire us and urge us to think outside the box for a radical transformation of our roles in the recovery stage of this pandemic. They’ve discussed about implementing different active learning strategies in virtual teaching , consolidating learning management system and delivering patient care in the virtual world. They offer many useful practical tips to adjust the sails during the winds of change. They show us that the possibilities are endless to facilitate learning in the virtual learning environment, if technology is our friend. They emphasize that it’s right time to move forward towards future that medical education has been awaiting for decades and the time for innovation is Now and only Now…!!

Reviewer Expertise:

NA

No decision status is available
